# Rat Strain and Housing Conditions Alter Oxidative Stress and Hormone Responses to Chronic Intermittent Hypoxia

**DOI:** 10.3389/fphys.2018.01554

**Published:** 2018-11-06

**Authors:** Brina Snyder, Phong Duong, Mavis Tenkorang, E. Nicole Wilson, Rebecca L. Cunningham

**Affiliations:** Department of Physiology and Anatomy, University of North Texas Health Science Center, Fort Worth, TX, United States

**Keywords:** hypothalamic–pituitary–adrenal axis, reproducibility, oxidative stress, corticosterone, ACTH, hyperphosphorylated tau

## Abstract

Sleep apnea has been associated with elevated risk for metabolic, cognitive, and cardiovascular disorders. Further, the role of hypothalamic–pituitary–adrenal (HPA) activation in sleep apnea has been controversial in human studies. Chronic intermittent hypoxia (CIH) is a rodent model, which mimics the hypoxemia experienced by patients with sleep apnea. Most studies of CIH in rats have been conducted in the Sprague Dawley rat strain. Previously published literature suggests different strains of rats exhibit various responses to disease models, and these effects can be further modulated by the housing conditions experienced by each strain. This variability in response is similar to what has been observed in clinical populations, especially with respect to the HPA system. To investigate if strain or housing (individual or pair-housed) can affect the results of CIH (AHI 8 or 10) treatment, we exposed individual and pair-housed Sprague Dawley and Long-Evans male rats to 7 days of CIH treatment. This was followed by biochemical analysis of circulating hormones, oxidative stress, and neurodegenerative markers. Both strain and housing conditions altered oxidative stress generation, hyperphosphorylated tau protein (tau tangles), circulating corticosterone and adrenocorticotropic hormone (ACTH), and weight metrics. Specifically, pair-housed Long-Evans rats were the most sensitive to CIH, which showed a significant association between oxidative stress generation and HPA activation under conditions of AHI of 8. These results suggest both strain and housing conditions can affect the outcomes of CIH.

## Introduction

There is a lack of consensus in the literature related to the basic scientific model of sleep apnea, chronic intermittent hypoxia (CIH). CIH has been reported to be both protective and damaging in subsequent stroke outcomes. Elevated mean arterial pressure, inflammation, oxidative stress, and cognitive impairment have been described in models of CIH (Cai et al., 2010; Smith et al., 2013; Rosenzweig et al., 2014; Briancon-Marjollet et al., 2016; Shell et al., 2016; Snyder et al., 2017), while other studies have reported lower oxidative stress and pre-conditioning effects of CIH (Zhen et al., 2014; Yuan et al., 2015). These divergent reports further complicate interpretation of the CIH animal model and consequently our understanding of sleep apnea (Navarrete-Opazo and Mitchell, 2014). The pivotal factors contributing to these dichotomous observations of CIH are most likely the frequency and severity of the intermittent hypoxia used in each study (Navarrete-Opazo and Mitchell, 2014). There appears to be a threshold in which studies using a more frequent normal room air to low oxygen cycle per hour, modeling the apnea/hypopnea index (AHI), result in damaging effects, while models with very slow air changes per hour report protective mechanisms.

In addition to differences in CIH protocols, prior studies have been conducted on various rat strains under different housing conditions. Our laboratory has exposed single-housed Sprague Dawley (Snyder et al., 2017), pair-housed Brown Norway (Wilson et al., 2018), and pair-housed Long-Evans (Snyder et al., 2018) rat strains to CIH with varying oxidative stress responses. Most CIH protocols have been performed on either Sprague Dawley or Wistar rat strains, but not the Long-Evans or Brown Norway rat strains. Generally, housing conditions are not reported (Ramanathan et al., 2005; Cai et al., 2010; Williams et al., 2015; Luneburg et al., 2016). Of the studies that include housing conditions, it is interesting to note that publications using single-housed Sprague Dawley rats report increased oxidative stress in response to CIH (Shell et al., 2016; Snyder et al., 2017; Li et al., 2018). Therefore, rat strain and housing conditions may be important variables.

The scientific community has recognized a number of factors exist which contribute to contradictory reports in many basic science models. In recognition of this, the National Institutes of Health (NIH) updated their mission of Rigor and Responsibility to improve reproducibility (Hewitt et al., 2017). Current variables recognized as having an impact on experimental outcomes are sex, age, weight, and current health status. Here, we present results supporting model strain and housing conditions as key components affecting the observed outcomes in our study using CIH. These results provide evidence that the variable, housing conditions, should be included in future studies.

## Materials and Methods

### Animals

Two outbred rat strains were used in this study – Sprague Dawley and Long-Evans. Adult male Sprague Dawley and Long-Evans rats (both strains 58–64 days old, 250–275 g body weight, Charles River) were housed in a temperature-controlled environment with the lights on a 12:12 h cycle. The Sprague Dawley rat strain is a non-aggressive rat strain, whereas the Long-Evans rat strain is more aggressive and active than Sprague Dawley rats (Adams and Blizard, 1987; Henry et al., 1993; Turner and Burne, 2014). Thus, we examined single-housing versus pair-housing in both rat strains. Upon arrival, animals were either housed individually or pair-housed with an unfamiliar rat of similar size and weight for the remainder of the experiment. Food and water were provided *ad libitum*. Animals were weighed each week during cage cleaning and at the end of testing. Final body weights were used for reporting purposes. All experiments were conducted according to National Institute of Health guidelines on laboratory animals and approved by the Institutional Care and Use Committee at UNT Health Science Center.

### Chronic Intermittent Hypoxia (CIH)

Hypoxic A-Chambers and OxyCycler A84XOV controllers were purchased from BioSpherix, Ltd. (Parrish, NY, United States). One week after arrival, rats were separated into either normoxic or CIH treatment groups with at least 10 animals per group. This resulted in eight treatment groups: single-housed Sprague Dawley normoxic (*n* = 13), single-housed Sprague Dawley CIH (*n* = 12), pair-housed Sprague Dawley normoxic (*n* = 17), pair-housed Sprague Dawley CIH (*n* = 16), single-housed Long-Evans normoxic (*n* = 12), single-housed Long-Evans CIH (*n* = 12), pair-housed Long-Evans normoxic (*n* = 12), and pair-housed Long-Evans CIH (*n* = 12). Home cages, each containing either single or pair-housed animals, were placed into each A-chamber for acclimation to the apparatus for 1 week at normoxic conditions (21% oxygen). Acclimation to the chambers was followed by CIH exposure for 7 days from 8 am to 4 pm during the light (sleep) phase. Our CIH protocol utilized 8-min cycles of low oxygen (10%) followed by reoxygenation (21%) for 8 h during the light phase to model an AHI 8 (Epstein et al., 2009; Snyder et al., 2018; Wilson et al., 2018). Specifically, nitrogen was injected into the chamber over a period of 5 min to reach a low oxygen concentration of 10%, followed by injection of oxygen over 3 min to return to and maintain normal room air concentrations (21%). For the remaining 16 h, animals were exposed to room air. To control for sleep deprivation, due to noises from the CIH apparatus, normoxic controls were housed under similar conditions but not administered hypoxia.

An additional group of single-housed Sprague Dawley normoxic (*n* = 3), single-housed Sprague Dawley CIH (*n* = 8), pair-housed Long-Evans normoxic (*n* = 4), and pair-housed Long-Evans CIH (*n* = 8) were exposed to a CIH protocol injecting nitrogen over a period of 3 min to reach an oxygen concentration of 10% and reoxygenation for 3 min to mimic an AHI 10 to compare results to previously published observations. Both AHI of 8 and 10 protocols result in oxygen nadirs of 10% lasting 75 s. One limitation of this CIH animal model for sleep apnea is that the nadir is longer than a typical apnea observed in patients with obstructive sleep apnea. However, the nadir duration is similar to the 15–60 s of hypoxemia experienced by obstructive sleep apnea patients (Dewan et al., 2015). This difference in nadir duration is primarily due to the size of the chambers that house the rats in their home cages and the amount of time needed for gas exchanges.

### Sample Collection

Between 08:00 and 10:00 on the morning following the final CIH exposure, which was during the first 2 h of the light phase, animals were anesthetized with isoflurane (2–3%) and sacrificed by decapitation, as previously described (Snyder et al., 2017). Trunk blood was collected in 7 mL EDTA tubes. The samples were then centrifuged at 2,000 × *g* for 10 min at 4°C. Plasma was removed and aliquoted for storage in microcentrifuge tubes at -80°C until assayed. Whole brains were immediately removed and flash frozen in PBS and coronally sliced to reveal brain nuclei of interest: the entorhinal cortex, and dentate gyrus (DG), CA1, and CA3 regions of the hippocampus. These nuclei were selected based on oxidative stress damage due to CIH that was observed in a previous publication (Snyder et al., 2017). Nuclei were dissected by micropunch as previously described (Snyder et al., 2017) and stored in microcentrifuge tubes at -80°C until homogenized for protein analysis.

### Advanced Oxidative Protein Products (AOPP) Assay

Plasma oxidative stress was assayed using Cell Biolabs, Inc. OxiSelect Advanced Oxidative Protein Products assay kit, according to our previously published protocol (Cunningham et al., 2011; Snyder et al., 2017, 2018). This kit measures the amount (uM) of all oxidized proteins in the sample relative to a known standard. Chloramine in the kit reacts with oxidized proteins to produce a color change which can be read at 340 nm. Assay results were reported as percent of control [individual value/(average of normoxic control values) × 100].

### Hormone Measurements

#### Corticosterone

Circulating nadir corticosterone was assayed using a commercially available competitive immunoassay (Corticosterone Mouse/Rat ELISA kit, RTC002R, BioVendor, Brno, Czechia), according to manufacturer’s instructions. Sensitivity of the assay was 6.1 ng/ml at the 2 SD confidence limit. The intra-assay coefficient of variation was 7.37% and the inter-assay coefficient of variation was 7.63%. Specificity of this assay is as follows: corticosterone (100%), cortisol (2.3%), aldosterone (0.3%), testosterone (<0.1%), progesterone (6.2%), and androsterone (<0.1%). Results are expressed as ng/ml.

#### Adrenocorticotropic Hormone

Plasma adrenocorticotropic hormone (ACTH) was assayed by double-antibody radioimmunoassay using ^125^I (hACTH Double Antibody RIA Kit, 07-106102, MP Biomedicals, Solon, OH, United States), according to manufacturer’s protocol. Samples were performed in duplicates and the assay was measured using a gamma counter (Cobra Auto-Gamma, LPS Biomedical Instrument Services, Redmond, WA, United States), with counting time of 3 min per sample at 80% efficiency. The intra-assay coefficient of variation was 5.45% and the inter-assay coefficient of variation was 7.30%. The specificity of the assay is as follows: ACTH^1-39^ (100%), ACTH^1-24^ (100%), hβ lipotropin (0.8%), hα lipotropin (0.1%), hβ endorphin (<0.1%), hα MSH (<0.1%), and hβ MSH (<0.1%). The following formula was used to determine the %B/B0:

$$[(CPM_{\mathrm{Sanple}}-CPM_{\mathrm{NSB}})/(CPM_{0\mathrm{Standard}}-CPM_{\mathrm{NSB}})]\times 100$$

in which CPM = counts per minute, NSB = non-specific binding (blank), 0 standard = total binding (B0). The %B/B0 for the unknowns were then plotted against the standard %B/B0 using a 4 parameter-log function to determine ACTH concentration (analysis). Results are expressed as ng/ml.

### Tissue Homogenization

Frozen tissue samples were thawed in 50 uL RIPA solution (Amresco) containing 3 uM phosphatase inhibitor (Sigma-Aldrich), 1 uM EDTA (Sigma Aldrich), and 1 uM dithiothreitol (Sigma-Aldrich) as previously described (Snyder et al., 2017). Protein quantification was assessed using a commercially available Pierce BCA Protein Assay Kit (Thermo Fisher) and absorbance read at 562 nm to determine sample volumes for further analysis. Samples were stored at -80°C.

### Immunoblotting

Equal volumes of tissue samples containing 20 uL protein were loaded into a Bio-Rad 4–20% polyacrylamide gel for electrophoresis at 25 mA, followed by overnight transfer onto a PDVF membrane at 60 mA. Transfer was verified by Ponceau S staining, the washed for 30 min in TBST. Membranes were blocked for 60 min with 5% non-fat milk in TBS-Tween (TBST) at room temperature. Membranes were then transferred to a 1% non-fat milk TBST solution containing specific primary antibody for tau phosphorylated at S202 (Abcam ab108387, 1:10,000) and incubated overnight at 4°C. Afterward, membranes were washed in 10 min increments for 30 min, and then incubated in 1% milk TBST secondary antibody solutions (goat anti-rabbit 1:10,000) at room temperature for 1 h. Protein bands were visualized using West Pico enhanced chemiluminescence detection assay (Thermo scientific) on an Syngene G:Box system using FlourChem HD2 AIC software. Membranes were then incubated in 1% non-fat milk TBST solutions containing primary antibody for GAP-DH (D16H11) XP (HRP conjugate) (Cell Signaling Technology #8884, 1:10,000) for 2 h at room temperature, followed by chemiluminescent visualization. Although prior studies have shown GAP-DH expression can be affected by hypoxic exposure, no significant differences due to CIH were observed in any of the brain regions examined. NIH ImageJ software (version 1.50i) was used to quantify band densitometry and values were normalized to GAP-DH values using the equation (mean gray value for protein/mean gray value of GAP-DH)^∗^100.

### Statistical Analysis

IBM SPSS (SPSS v. 23, IBM, 2015) was used for statistical analysis. Three-way ANOVA was used to test for significant interactions between strain, housing condition, and hypoxic exposure. Fisher’s LSD was used for *post hoc* analysis. One-way ANOVAs were used to analyze the effect of hypoxia within individual brain regions. Results are shown as mean ± SEM. Statistical significance for all measurements was at *p* ≤ 0.05.

## Results

### Strain Differences in Chronic Intermittent Hypoxia-Induced Oxidative Stress

**FIGURE 1:**
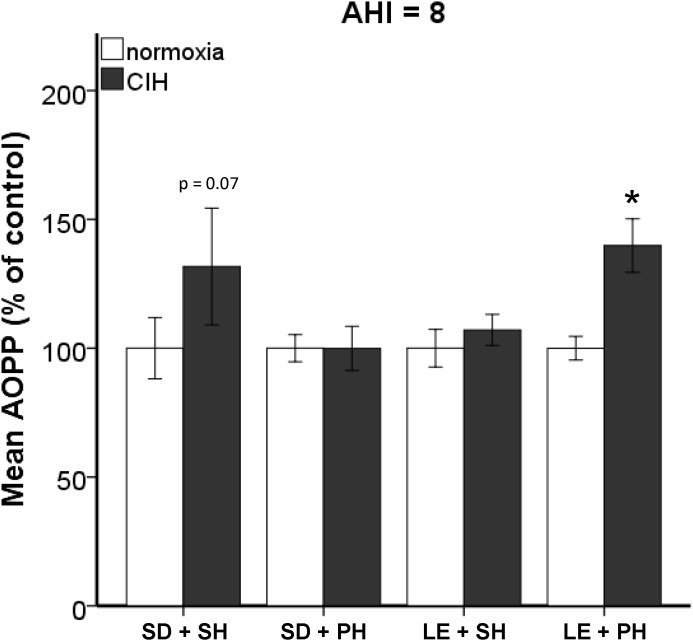
Strain and housing conditions alter oxidative stress response to chronic intermittent hypoxia (CIH) modeling an apnea/hypopnea index (AHI) of 8. Oxidative stress was measured in plasma using Advanced Oxidative Protein Products (AOPP) assay. Long-Evans pair-housed rats (LE + PH) exhibit significantly higher oxidative stress when exposed to chronic intermittent hypoxia than normoxic controls. In Sprague Dawley single-housed rats (SD + SH), oxidative stress was not increased by CIH. No significant differences in oxidative stress were observed in Sprague Dawley pair-housed (SD + PH) or Long-Evans single-housed (LE + SH) rats. Results are reported as mean ± SEM (percent of normoxic control values), ^∗^ compared to normoxic control.

**FIGURE 2:**
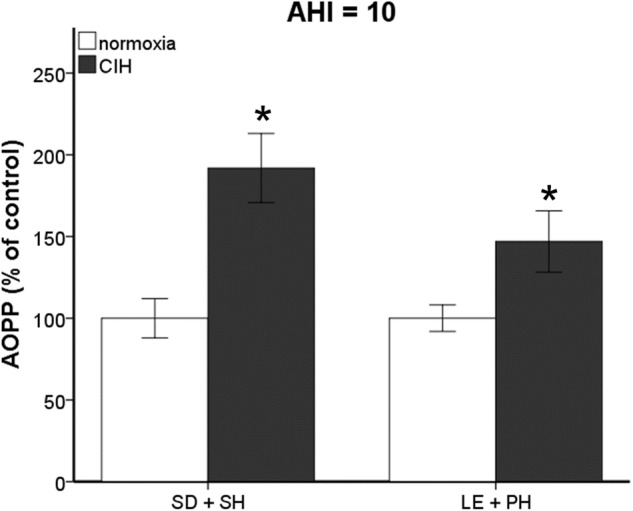
Increasing the frequency of air exchanges during chronic intermittent hypoxia (CIH) results in significant increases in oxidative stress. Advanced Oxidative Protein Products (AOPP) is significantly elevated in Sprague Dawley single-housed (SD + SH) and Long-Evans pair-housed (LE + PH) rats following 7 days exposure to CIH at an apnea/hypopnea index (AHI) 10. Results are reported as mean ± SEM (percent of normoxic control values), ^∗^ compared to normoxic control.

Previously, we published that exposure to CIH (AHI 10) induced oxidative stress in plasma and brain regions associated with neurodegeneration in single-housed Sprague Dawley male rats (Snyder et al., 2017). To investigate if this observation is maintained across strain and housing conditions, both Sprague Dawley and Long-Evans male rats were housed singly or in pairs and then exposed to 7 days mild CIH (AHI 8) or normoxic conditions. Pair-housed Sprague Dawley rats and single-housed Long-Evans rats did not experience an increase in oxidative stress following 7 days of CIH (Figure 1). However, a significant interaction between strain, housing, and hypoxia (*F*_1,89_ = 5.842; *p* < 0.05) was observed. A significant elevation of oxidative stress was observed in pair-housed Long-Evans rats (139.84 ± 32.87%) (Figure 1) following CIH exposure. Unlike what is observed at AHI 10 (Figure 2), single-housed Sprague Dawley rats did not have a significant increase in oxidative stress due to CIH at AHI 8. At AHI 10, there were no significant differences between strains in the magnitude of the oxidative stress response to CIH (*F*_1,14_ = 2.53, *p* = 0.13). Interestingly, under normoxic conditions Long-Evans rats exhibit higher circulating basal oxidative stress (312.3 ± 96.41 uM) than Sprague Dawley rats (219.69 ± 83.63 uM), regardless of housing condition (*F*_1,51_ = 14.00, *p* < 0.05). These observations suggest Long-Evans rats are more sensitive to hypoxic insults than Sprague Dawley rats, which may be due to higher circulating basal oxidative stress in Long-Evans rats. These results indicate housing conditions can impact susceptibility to oxidative stress differently between strains.

### Strain Differences in Phosphorylated Tau Induced by Chronic Intermittent Hypoxia

**FIGURE 3:**
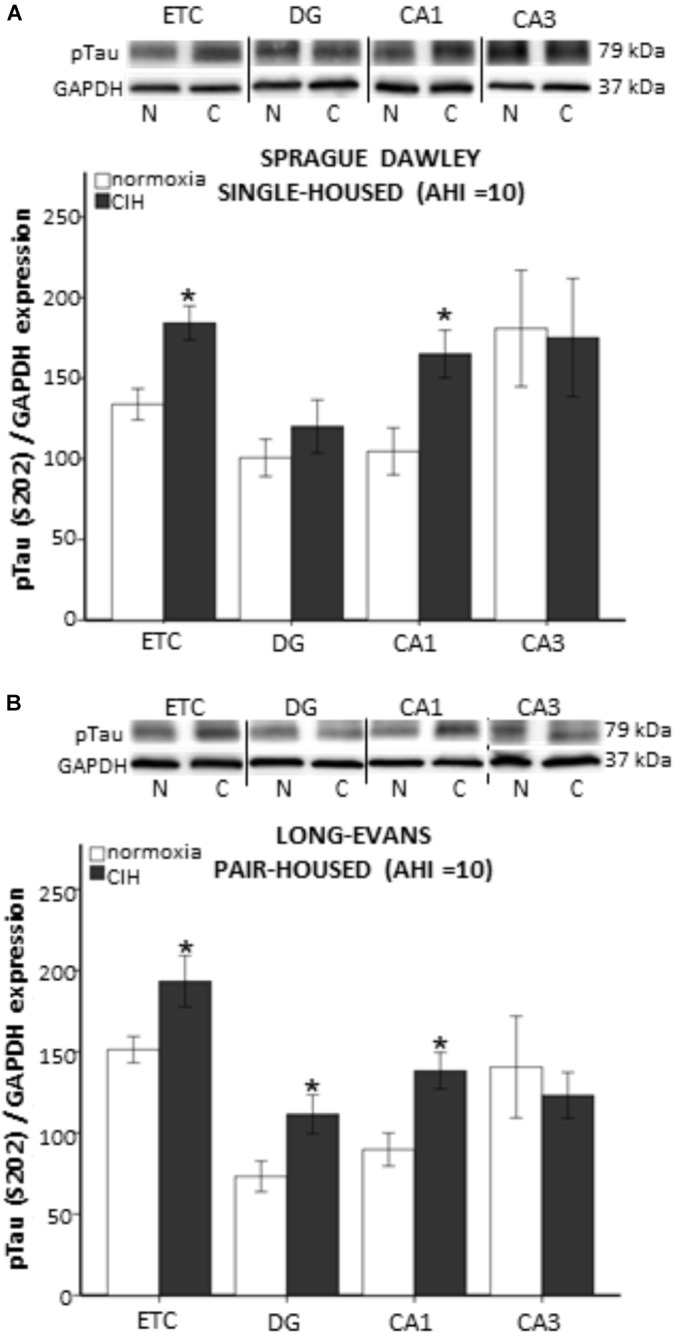
Evidence of tau tangles are present in memory-associated brain nuclei following 7 days of chronic intermittent hypoxia (CIH) at apnea/hypopnea index (AHI) = 10. Tissue homogenate from each brain region investigated was run on separate gels for western blot analysis. Representative images from each membrane are grouped together and displayed in **(A,B)** for each strain of rat. Western blot analysis of hyperphosphorylated tau (p-tau) was performed on tissue homogenate from the entorhinal cortex (ETC), and dentate gyrus (DG), CA1, and CA3 regions of the hippocampus of normoxic (N) and CIH (C) exposed male rats. Evidence of CIH contribution to tau tangle accumulation was observed in a strain dependent manner. **(A)** Single-housed Sprague Dawley rats exhibit significantly elevated p-tau in the ETC and CA1 after CIH exposure. **(B)** Pair-housed Long-Evans rats exhibit significantly elevated p-tau in the ETC, as well as in the DG, and CA1 regions of the hippocampus. Results are reported as mean ± SEM (p-tau/GAP-DH expression), ^∗^ compared to normoxic control.

Chronic intermittent hypoxia has been associated with cognitive impairments in rodent models of sleep apnea (Xu et al., 2004; Cai et al., 2010; Smith et al., 2013; Sapin et al., 2015; Snyder et al., 2018). To determine if strain or housing impact brain regions involved in memory pathways, we assessed the presence of tau hyperphosphorylated at serine 202 (p-tau), an indicator of tau tangle accumulation. The presence of mitochondrial oxidative damage has been reported concurrently with tau tangle accumulation in post-mortem temporal lobes of persons with preclinical stages of Alzheimer’s disease (Terni et al., 2010). Because we were most interested in processes that may be impacted by oxidative stress generation, we only used single-housed Sprague Dawley rats and pair-housed Long-Evans rats, which exhibited CIH-induced plasma oxidative stress. Although we did not observe any differences in p-tau protein expression in the entorhinal cortex (ETC) or hippocampal structures at AHI 8 in any of our groups (data not shown), strain differences in the p-tau protein expression were observed under CIH condition of AHI 10. Significantly more p-tau in the ETC (*F*_1,5_ = 12.457; *p* < 0.05) and CA1 (*F*_1,7_ = 8.543; *p* < 0.05) hippocampal brain regions was observed in single-housed Sprague Dawley rats exposed to CIH at AHI 10 than their normoxic counterparts (Figure 3A). Similar to our observations with AOPP, pair-housed Long-Evans rats appear to be more sensitive to CIH exposure, with more p-tau protein expression in the dentate gyrus (DG) and CA1 (*F*_1,8_ = 8.739; *p* < 0.05) of the hippocampus (*F*_1,9_ = 5.340; *p* < 0.05), as well as in the ETC (*F*_1,8_ = 8.800; *p* < 0.05), following CIH exposure at AHI 10 (Figure 3B).

### Strain Differences in Hypothalamus–Pituitary–Adrenal (HPA) Axis Activation

#### HPA Hormone Differences

Corticosterone and its releasing hormone, ACTH, can be affected by housing conditions, and corticosterone may contribute to oxidative stress burden (Zafir and Banu, 2009; Spiers et al., 2015; Solanki et al., 2017). To determine if strain type or housing conditions produces a differential response to CIH at AHI 8, circulating nadir corticosterone and ACTH were assayed. A significant interaction between strain and CIH at an AHI 8 (*F*_1,63_ = 5.988, *p* < 0.05) was observed in ACTH levels, with a main effect of both strain (*F*_1,63_ = 75.735, *p* < 0.05) and CIH (*F*_1,63_ = 13.475, *p* < 0.05) on plasma ACTH levels (Table 1). Under normoxic conditions, Long-Evans rats had significantly higher ACTH than Sprague Dawley rats. In pair-housed Long-Evans rats, CIH significantly decreased ACTH compared to normoxic ACTH levels. Following CIH at AHI 10, both single-housed Sprague Dawley rats (*F*_1,8_ = 4.96, *p* < 0.05) and pair-housed Long-Evans rats (*F*_1,14_ = 29.33, *p* < 0.05) had significantly lower ACTH than normoxic controls (Table 1). Further, ACTH following AHI 10 was significantly lower than after AHI 8 in both strains (Sprague Dawley single-housed: *F*_1,14_ = 11.85, *p* < 0.05; Long-Evans pair-housed: *F*_1,12_ = 13.33, *p* < 0.05; Table 1).

**Table 1:** Hypothalamic–pituitary–adrenal axis hormone concentrations are affected by strain and hypoxia at AHI 8, with an interaction between housing and hypoxia.

Strain	Housing	Hypoxia	*n*	ACTH (ng/ml)	SD	Significance	CORT (ng/ml)	SD	Significance
Sprague Dawley	Single	Norm	8	156.33	46.43		34.93	24.66	
		CIH (8)	9	143.82	17.48		37.96	18.21	
		CIH (10)	7	88.76	44.09	^∗^+	23.61	22.68	
	Pair	Norm	9	148.57	46.20		33.50	15.30	
		CIH (8)	12	162.41	44.47		22.05	11.97	
Long-Evans	Single	Norm	8	381.45	75.20		41.71	23.84	
		CIH (8)	8	285.05	122.01		41.50	14.91	
	Pair	Norm	7	371.53	69.86		51.12	12.01	
		CIH (8)	7	263.52	54.57	^∗^#	27.41	18.00	^∗^#
		CIH (10)	7	117.99	70.60	^∗^+	9.78	9.75	^∗^+


*Male Long-Evans rats have significantly higher circulating nadir corticosterone (CORT) and ACTH than Sprague Dawley rats. Results are reported as mean ± SD (ng/ml), ^∗^compared to normoxic control; ^+^compared to AHI 10; ^#^interaction with housing; statistical significance was set at *p* ≤ 0.05.*

**FIGURE 4:**
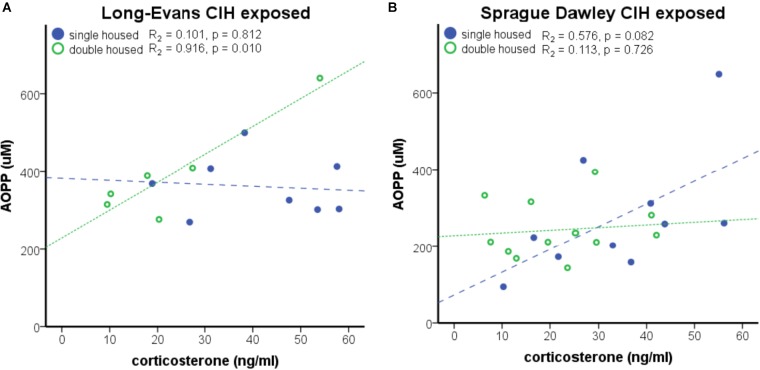
Strain differences are apparent in the positive association of nadir corticosterone and oxidative stress under chronic intermittent hypoxia (CIH). **(A)** A significant positive correlation between nadir corticosterone and oxidative stress, as measured by Advanced Oxidative Protein Products (AOPP), was observed in Long-Evans pair-housed male rats following 7 days of CIH exposure at apnea/hypopnea index (AHI) = 8. **(B)** AOPP and corticosterone are not significantly associated in Long-Evans single-housed or Sprague Dawley male rats.

An interaction between housing and CIH (AHI 8) on corticosterone was also observed (*F*_1,68_ = 6.015, *p* < 0.05). Similar to ACTH, Long-Evans pair-housed rats exposed to CIH had significantly lower corticosterone than their normoxic counterparts (Table 1). Since prior studies have shown that corticosterone can increase oxidative stress (Zafir and Banu, 2009; Spiers et al., 2015), we wanted to determine if there was a relationship between corticosterone and CIH-induced oxidative stress. Therefore, we investigated this association on our treatment groups (single-housed Sprague Dawley and pair-housed Long-Evans rats) that showed an elevation of oxidative stress in response to CIH. We observed a positive association between corticosterone and oxidative stress only in Long-Evans pair-housed rats exposed to CIH (Figure 4A). Neither the Long-Evans single-housed rats nor either of the Sprague Dawley rat cohorts exhibited an association between oxidative stress and nadir corticosterone following CIH exposure (Figures 4A,B).

Following CIH at AHI 10, Long-Evans pair-housed rats exhibited lower corticosterone than normoxic controls (*F*_1,14_ = 54.53, *p* < 0.05; Table 1), similar to what was observed with ACTH. Additionally, exposure to the higher AHI = 10 resulted in further suppression of corticosterone than AHI 8 did (*F*_1,12_ = 4.90, *p* < 0.05; Table 1). Unlike what was observed with ACTH, there were no significant differences in corticosterone levels in the Sprague Dawley single-housed rats following AHI 10 (*F*_1,8_ = 0.377, *p* < 0.05; Table 1). Additionally, an association between oxidative stress and nadir corticosterone was not present in either strain exposed to AHI 10 (data not shown). This indicates a strain difference in sensitivity to hypoxia that is dependent on social housing environment and frequency of air exchanges.

#### Body Weight Changes due to Strain and Housing

**FIGURE 5:**
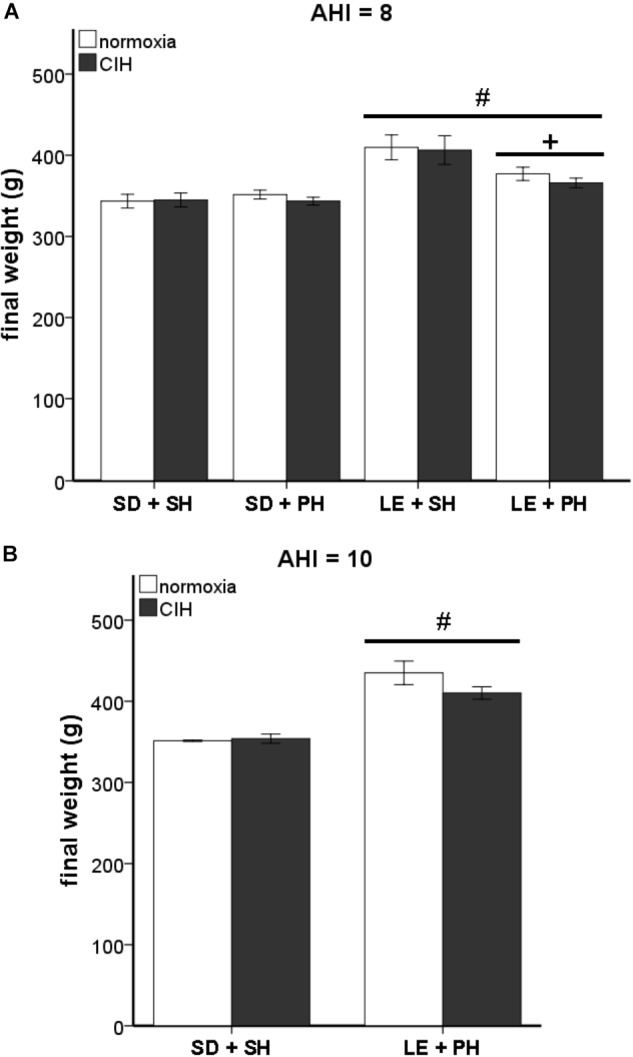
Differences in final weight are not due to chronic intermittent hypoxia (CIH). Long-Evans rats were significantly heavier than Sprague Dawley rats, but no differences due to CIH at an apnea/hypopnea index (AHI) = 8 or 10 were observed. **(A)** Male Long-Evans rats weigh significantly more than male Sprague Dawley rats, regardless of housing conditions. Social housing conditions results in lower weight than single-housed Long-Evans rats. CIH at AHI 8 did not cause any significant differences in weight in either strain. **(B)** Long Evans rats weigh more than Sprague Dawley rats, even at AHI 10. CIH at AHI 10 does not alter weight in either Sprague Dawley rats or Long-Evans rats. Results are reported as mean ± SEM (g), # compared to Sprague Dawley strain, + compared to single-housed.

Activation of the HPA system can influence body weight (Faraday et al., 2005; Bhatnagar et al., 2006; Page et al., 2016). In our study, a statistically significant interaction between strain and housing conditions on final weight was observed at an AHI 8 (*F*_1,93_ = 7.597; *p* < 0.05). Analysis of the main effects revealed a significant difference in weight between the two strains of rats (*F*_1,93_ = 41.746, *p* < 0.05), with Long-Evans rats weighing more than Sprague Dawley rats (Figure 5A). This strain difference was maintained between Sprague Dawley and Long-Evans rats when they were exposed to an AHI 10 (*F*_1,17_ = 55.673, *p* < 0.05; Figure 5B). A significant difference in housing (*F*_1,93_ = 5.075, *p* < 0.05) was observed in Long-Evans rats in which pair-housed Long-Evans rats weighed less than the single-housed Long-Evans rats (Figure 5A). The housing conditions of Sprague Dawley rats did not affect weight in this study. Additionally, exposure to CIH at either AHI 8 or 10 did not affect the final weight of any of the treatment groups or correlate with oxidative stress measurements (Table 2).

**Table 2:** Weight is not significantly associated with oxidative stress in either Sprague Dawley or Long-Evans rats following CIH at AHI 8 or 10.

Strain	Housing	Hypoxia	*r*^2^	*p*-Value
Sprague Dawley	Single	Norm	0.08	*0.43*
		CIH (8)	0.12	*0.39*
		CIH (10)	0.30	*0.15*
	Pair	Norm	0.01	*0.77*
		CIH (8)	0.01	*0.75*
Long-Evans	Single	Norm	0.21	*0.13*
		CIH (8)	0.02	*0.65*
	Pair	Norm	0.28	*0.07*
		CIH (8)	0.17	*0.24*
		CIH (10)	0.28	*0.18*


## Discussion

Our current experiment utilized a protocol modeling an AHI 8 or 10 to examine the effects of 7-day CIH treatment on two different strains of rats, Sprague Dawley and Long-Evans, housed either singly or in pairs. Prior studies have indicated substantial physiological changes occur at this early time point which contributes to the overall detrimental effects of CIH. For example, there is an elevation of mean arterial pressure and heart rate (Knight et al., 2011), activation of the HPA axis (Ma et al., 2008), and an elevation of oxidative stress and inflammation in both plasma and brain regions susceptible to neurodegenerative processes (Snyder et al., 2017). Behavioral studies have provided evidence that strain differences and social interaction via housing conditions can affect the outcome of many studies (Bielajew et al., 2002; Faraday, 2002; Faraday et al., 2005; Tan et al., 2009; Konkle et al., 2010; Turner and Burne, 2014). Consideration of these factors allows for a more robust understanding of the mechanisms of disease and improves therapeutic outcomes. To investigate how strain and housing conditions can affect reported outcomes of CIH, we elected to quantify markers of HPA activation, plasma oxidative stress, and hyperphosphorylated tau (p-tau), an indicator of neurodegenerative processes.

Interestingly, only one study has examined the differences between Sprague Dawley and Long-Evans rats in response to oxygen exposure. Unlike our study using intermittent low oxygen levels, Chrysostomou et al. (2010) examined hyperoxia (75% oxygen) exposure for 14 days and found Long-Evans rats were more sensitive to oxygen than Sprague Dawley rats, resulting in increased cell death and astrocyte upregulation. Consistent with these observations, significant differences in oxidative stress and tau tangles in a strain dependent manner were observed in this study (Figures 1–3). Pair-housed Long-Evans rats exhibited an increase in oxidative stress similar to what was previously observed in the single-housed Sprague Dawley rats at a slightly higher AHI (Snyder et al., 2017). The single-housed Sprague Dawley rats used in this study did not show significant increase in oxidative stress under CIH conditions at an AHI 8, but did exhibit elevated oxidative stress at the higher hypoxic frequency of AHI 10, confirming our previous studies at AHI 10 (Snyder et al., 2017). Thus, Long-Evans rats in group housing conditions may be more sensitive to oxidative insults than Sprague Dawley rats. Interestingly, neither the pair-housed Sprague Dawley nor the single-housed Long-Evans rats exhibited an oxidative stress response to early CIH at AHI 8. These results suggest an interaction between genetic differences in the rat strains and housing conditions influences oxidative stress activation. These parameters should be considered when investigating mechanisms contributing to oxidative stress.

In our previous study (Snyder et al., 2017), 7 days CIH at an AHI 10 elevated plasma oxidative stress and oxidative stress within brain regions susceptible to early neurodegenerative mechanisms. Evidence exists in the literature supporting the involvement of elevated oxidative stress on p-tau generation (Terni et al., 2010; Schulz et al., 2012). To investigate how elevated plasma oxidative stress due to CIH might reflect neuropathological changes, p-tau was quantified in the ETC and subregions of the hippocampus. Although we saw no effects on p-tau at AHI 8, p-tau was found to be significantly elevated in the ETC and CA1 subregion of the hippocampus in both pair-housed Long-Evans rats and single-housed Sprague Dawley rats exposed to CIH at AHI 10. Additionally, pair-housed Long-Evans rats were observed to express p-tau in the dentate gyrus subregion of the hippocampus, whereas single-housed Sprague Dawley rats did not. This is consistent with data supporting Long-Evans rats may be more susceptible to changes in oxygen than Sprague Dawley rats. Therefore, the p-tau observed in our study may be associated with the oxidative stress response to CIH. Of note, the increase in p-tau protein expression at AHI 10 coincided with significant HPA dysregulation, suggesting the HPA axis may also be involved in tau tangles. Indeed, the study by Carroll et al. (2011), demonstrates that acute administration of corticosterone reduced p-tau, whereas chronic stress for 1 month resulted in elevated p-tau. Our results are consistent with changes due to chronic HPA activation.

In both humans and rodents, social interaction and environmental stress impact disease risk (Saxton et al., 2011). Activation of the HPA axis occurs under stressful scenarios. Under acute stress conditions, elevated peak corticosterone or ACTH is indicative of an HPA activation (Bhatnagar et al., 2006). However, under chronic stress, decreased HPA hormones have been observed (Bielajew et al., 2002; Rich and Romero, 2005). Maintaining homeostatic HPA hormones is necessary in maintaining sleep architecture (Karatsoreos et al., 2011; Machado et al., 2013). Corticosterone and ACTH fluctuate in diurnal patterns that mirror each other, with low concentrations occurring at the beginning of the sleep phase (Chacon et al., 2005; Caride et al., 2010). Although our samples were collected during the nadir phase, the further suppression of HPA hormones as the AHI increased indicates CIH was a chronic stressor resulting in dysregulation of the HPA axis. The floor effect of corticosterone and elevated oxidative stress at AHI 10, highlights the potential sensitivity of Long-Evans rats to intermittent hypoxia. Similar to our results, prior studies found basal ACTH was not altered in single-housed Sprague Dawley rats exposed to 7 days CIH (AHI 10), but was more reactive when those same rats were subjected to a subsequent stressor (30 min immobilization) (Ma et al., 2008). Sprague Dawley rats housed individually consistently present significantly greater HPA responses than Sprague Dawley rats in group housing, suggesting socialization desensitizes Sprague Dawley rats to stress (Sharp et al., 2002).

The observed strain difference in ACTH, in which Long-Evans rats have higher ACTH than Sprague Dawley rats, suggests our measurements of the HPA axis are in agreement with existing publications (Sarrieau et al., 1998; Faraday et al., 2005; Ma et al., 2008). Although previous publications reported a difference in corticosterone between Sprague Dawley and Long-Evans rats, there were no basal differences in corticosterone due to strain or housing in this study. We did observe differences in nadir ACTH and corticosterone at AHI 8 and 10 between strains that are consistent with an HPA axis response to chronic stress (Table 1). The suppression in ACTH and corticosterone observed in the single-housed Sprague Dawley rats and the pair-housed Long-Evans rats may be indicative of HPA activation resulting in either elevated peak hormone levels or dysregulation of the diurnal cycle. Future studies which collect samples at peak times (at the beginning of the active phase) will help determine which scenario is accurate. Regardless, nadir corticosterone levels were positively correlated with oxidative stress only in the Long-Evans pair-housed rats at AHI 8, which was the only group to show CIH-induced oxidative stress at this AHI. These results suggest a relationship between activation of the HPA axis and oxidative stress generation. However, this relationship is no longer evident upon more intense AHI. Therefore, it possible that an AHI 8 induces an incomplete suppression of the HPA axis, suggesting AHI 8 is a milder chronic stressor than AHI 10.

The 2011 recommendation by the National Research Council for the care and use of laboratory animals is that social animals, such as rats, are to be housed in pairs or as a group (National Research Council (US) Committee for the Update of the Guide for the Care and Use of Laboratory Animals, 2011). This recommendation was based on prior studies using Sprague Dawley and Wistar rat strains that found decreased stress responses in rats housed in groups of three to four male rats/cage (Klir et al., 1984; Sharp et al., 2002). Similarly, our results show that housing conditions did not adversely affect Sprague Dawley male rats, under normoxic conditions. No differences in HPA hormones or final body weights were found in either individually or pair-housed rats, consistent with prior reports in Sprague Dawley male rats (Turner and Burne, 2014) (Figures 4, 5). However, not all rat strains respond the same to housing conditions. Group housing in Long-Evans rats has been associated with increased anxiety and reduced body weight (Scheufele et al., 2000; Bean et al., 2008). Similarly, our results showed male Long-Evans rats were adversely affected by pair-housing, as evidenced by decreased final body weights, regardless of hypoxic exposure (Figure 5). It has been proposed that this strain difference could be due to the level of aggression displayed by the different rat strains. For example, the Sprague Dawley rat strain is a non-aggressive rat strain, whereas the Long-Evans rat strain is more aggressive (Adams and Blizard, 1987; Henry et al., 1993). Henry (1992) and Henry et al. (1993) found that group housing of unfamiliar adult Long-Evans rats, and not Sprague Dawley rats, resulted in a prolonged activation of the stress-response and the inability to establish a stable dominance hierarchy. Indeed, we observed increased aggressive behaviors [attacks, threats, aggressive mounts, boxing, and dominance postures (Cunningham and McGinnis, 2006)] by Long-Evans males and not in Sprague Dawley males (data not shown). Therefore, stress, as evidenced by body weight loss, may underlie the observed differences in Sprague Dawley and Long-Evans rat strains to CIH.

**FIGURE 6:**
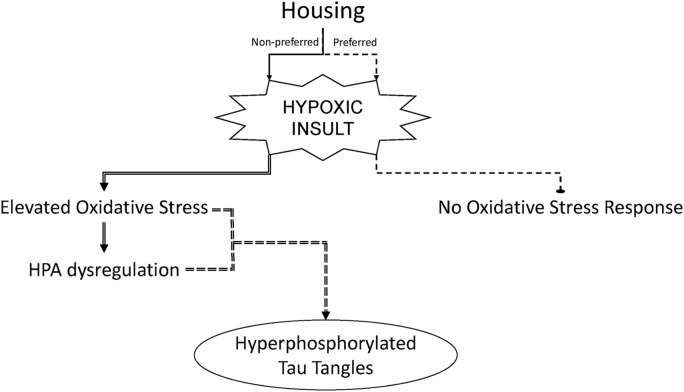
Strain housing preferences which contribute to activation of the hypothalamic–pituitary–adrenal (HPA) axis can sensitize male rats to hypoxia-induced oxidative stress and hyperphosphorylation of tau in the brain. Experimental outcomes of chronic intermittent hypoxia in male rats may be dependent upon the preferred housing condition of the rat strain used.

These results suggest that mechanisms which render an organism susceptible to oxidative stress insults may also impair the HPA axis. Elevated oxidative stress as well as HPA dysregulation may, in turn, contribute to mechanisms which initiate neurodegenerative processes, such as tau tangles (a key neuropathology characteristic of Alzheimer’s disease) (Figure 6). The results may not be immediately observable under non-stressful conditions, but manifest with the addition of a psychological or physiological stressor. A similar phenomenon is observed in clinical populations who experience chronic life stressors or illness and are subsequently exposed to an additional injury or infection (Rao, 2009; Seib et al., 2014; Duric et al., 2016; Jacob et al., 2018). They often succumb more rapidly and have lingering health concerns compared to individuals with less stress-response activation. Therefore, sleep apnea mechanisms may be additive and pose the highest risk to individuals with additional physiological or psychological stress.

Differences in strain response to CIH were observed in oxidative stress and corticosterone/ACTH measurements under different housing conditions and at different AHI levels. Although the differences between AHI 8 and AHI 10 were small, they were significant between strains and housing conditions. These results underscore the need for housing conditions to be included with strain reporting, especially in the investigations of any stressful stimuli, such as CIH. Factors which affect the HPA axis may influence the outcome of these types of studies. This study may shed light on discrepancies found between labs that use different animal strains and housing conditions, as well as guide future experimental design choices when selecting an animal model.

## Author Contributions

BS wrote the paper, collected the data, and performed the analysis. PD edited the methods, collected the data, and performed the analysis. MT and EW collected the data and performed the analysis. RC conceived and designed the analysis, edited the paper, and is the primary investigator.

## Conflict of Interest Statement

The authors declare that the research was conducted in the absence of any commercial or financial relationships that could be construed as a potential conflict of interest.
